# How to Create a Hebrew Reader? *Olam Katan* (1901–1904) and the Young Hebrew Reading Public

**DOI:** 10.1007/s10583-022-09520-w

**Published:** 2022-12-13

**Authors:** Agnieszka Jagodzińska

**Affiliations:** Taube Department of Jewish Studies, University of Wrocław, Wrocław, Poland

**Keywords:** Jewish children’s press, Jewish children’s literature, Jewish education, Hebrew revival, Zionism

## Abstract

*Olam Katan* [‘Small world’] was the first illustrated Hebrew periodical for Jewish children, published first in Vienna (1901–1902), then in Cracow (1902–1904). Although the periodical reached three continents, the overwhelming majority of its readers were located in Eastern Europe. This article analyses the editors’ efforts to create an attractive yet educational magazine for young readers as a part of a much bigger enterprise: raising a generation of Hebrew users and contributing to the revival of Hebrew. Through a content analysis of the periodical, I reconstruct not only the editors’ strategy to reach children and young readers but also the obstacles they faced in the process. I also seek to determine what the role of adults was in the process of children’s Hebrew education in general, and in mediating the content of this Hebrew periodical in particular.

## Introduction

In the years around 1900, the Hebrew language was not commonly spoken or understood by Jewish children in Eastern Europe. Although it had remained central to Jewish religious practice and to many aspects of Jewish existence, European Jews spoke other languages in their everyday life: Yiddish, Judeo-Spanish, or—due to intensifying acculturation during the nineteenth century—local non-Jewish languages like German, Polish, and Russian. Hebrew had been a part of traditional education, but it had been taught via sacred texts and not as a living language of everyday life. The revival of Hebrew as a spoken language is generally associated with Zionism (the Jewish national movement), which aimed to restore Hebrew as the national language of the Jews. This, however, could not have been achieved without the earlier legacy of the Haskalah (the Jewish Enlightenment), which renewed Hebrew as the language of Jewish secular literature and press, laying the foundation for its further development.^1^ With Zionism’s rising popularity, which peaked after the first Zionist Congress in Basel in 1897, propagators of the Hebrew revival sought ways to reintroduce Hebrew as the vernacular language of the Jews.

This article studies one such attempt, namely the publication of the first illustrated Hebrew periodical for young readers, *Olam Katan: ‘iton shevu’i metsuyar li-vene ha-ne’urim*^2^ [‘Small world: a weekly illustrated paper for the young’]. This weekly was created by Warsaw-based editors Avraham Leib Shalkovich (aka Ben-Avigdor), a writer and the founder and owner of the renowned Tushiyah Publishing House, and his brother-in-law, Shemu’el Leib Gordon (aka Shalag), a writer and educator.^3^*Olam Katan* was printed first in Vienna (from 1901) and later in Cracow (1902–1904). Although there have been studies of various efforts to revive Hebrew, the majority of them tend to focus on Palestine (Erets Yisra’el) and not Eastern Europe.^4^ Therefore, the East-European contribution to the Hebrew revival is still insufficiently recognised and merits closer scholarly attention.

In this article, I analyse the editors’ efforts to create an attractive, pleasant, yet educational magazine for young readers which, I claim, was part of a much bigger enterprise: raising a generation of Hebrew users and future supporters of the Zionist cause. Through a content analysis of the periodical, I reconstruct the editors’ strategy to reach children and young readers, and the obstacles they faced in the process. I also seek to determine what the role of adults was in the process of children’s Hebrew education in general, and in mediating the content of this Hebrew periodical in particular.

## Early Hebrew Press for Jewish Children

Hebrew periodicals for children appeared quite late—at the end of the nineteenth century.^5^ It was more than 100 years after similar publications for a general young audience were printed for the first time in Western Europe, and a few decades after periodicals for Jewish children in other languages appeared (Ofek, 1979, p. 344). There were a few reasons for this lengthy delay. First and most obviously, Hebrew was not—as mentioned above—the vernacular language of Jews. In fact, Hebrew literature for children started with “no natural reading public” (Shavit, 1996, p. 788), and so did Hebrew press for children. Secondly, Hebrew education was heavily influenced by gender differentiation: until reforms of the traditional system of Jewish schooling, studying Hebrew was a part of boys’, and not girls’, curriculum.^6^ Thirdly, the popularity of Zionism, which grew from the end of nineteenth century, was yet another factor explaining the emergence of Hebrew children’s periodicals at this time.

The emergence of the Hebrew children’s press needs to be understood in geographical as well as chronological context. Uriel Ofek noted that the first Hebrew periodical for children, *Olam Katon*, appeared in Palestine; it was edited by Eli’ezer Ben-Yehudah, popularly known as “the father of modern Hebrew”,^7^ and his associates. It came out in Jerusalem in 1893 as a supplement to another periodical for adults. In Ofek’s opinion, the right time to create a periodical for young readers was only after Hebrew-language children’s literature established “a natural habitat” (Ofek, 1979, pp. 344–345). However, as Zeev Gries has correctly observed, the number of Jewish children in Palestine remained quite small until the Second Aliyah,^8^ and if one was to search for a significant young Hebrew-reading audience at the beginning of the twentieth century, the right address was still Europe, and not Palestine (Gries, 2007, p. 185). The fact that Eastern European Jewish children had to wait for a periodical longer than their peers in Palestine can be easily explained by the political situation in Russia and the Kingdom of Poland, where development of the press in Jewish languages was generally impeded throughout most of the nineteenth century.

The chronologically second Hebrew periodical for the young audience, but the first one printed outside Palestine, was *Gan Sha’ashu’im* [‘Garden of amusement’^9^]. It was published as a weekly in 1899–1900 in Lyck—then a town in East Prussia, very close to its border with the Kingdom of Poland and Russia, now Ełk in present-day Poland. Its editor was Avraham Mordechai Piurko, a teacher and author of publications for children. Contrary to the message suggested by its title, *Gan Sha’ashu’im* was more a tool of education and propaganda than a form of entertainment (see also Ofek, 1979, pp. 352). The periodical perfectly illustrates Zohar Shavit’s observation that “the existence of literature for pure amusement was unthinkable in terms of Jewish culture” (Shavit 1996, p. 784). Piurko’s editorial strategy and his approach to children’s needs were quite conservative. Texts written in a heavy and elaborate style were not necessarily appealing to a young audience, and the *nikud*—a system of vocalization, often added in contemporary children’s literature to help young readers decode and comprehend the text—did not appear on a regular basis (Ofek, 1979, pp. 352–353).^10^ While *Olam Katon* published only 7 issues, *Gan Sha’ashu’im* was discontinued after the 85th issue.^11^

*Olam Katan*, the periodical which I discuss in this article, was the third Hebrew children’s magazine but the first illustrated one: neither *Olam Katon* nor *Gan Sha’ashu’im* had any images. Compared to its predecessors, *Olam Katan* proved to be more successful in terms of the time span of the publication: it was printed from 1901 to 1904, and the total number of issues reached 175.^12^ But most importantly, it was the first children’s periodical whose editors really paid attention to children’s needs and tried to respond to them. Ben-Avigdor and Gordon created a magazine that educated Jewish children as Hebrew readers using a format that was both textually and visually attractive. It should be emphasized that, although created in Eastern Europe and mostly distributed here, *Olam Katan* received support from the initiators of the Hebrew revival in Palestine, both adults and children (see Even-Zohar Basmat, 2010, pp. 45–46).

## How to Create a Hebrew Reader?

While discussing the case of *Olam Katan*, it is important to remember that it was not an isolated endeavour undertaken by publisher Ben-Avigdor, but rather was a part of his bigger vision of propagating Hebrew culture. Jewish historian Azri’el Natan (Nosn) Frenk characterized the situation Ben-Avigdor found himself in, as a publisher of Hebrew literature, as follows: “If there are no Hebrew readers, you have to create them” (Frenk, 1916, p. 18).^13^ But how does one create a Hebrew reading public? One strategy was to make Hebrew literature as linguistically, thematically and financially accessible as possible, and Ben-Avigdor achieved this goal. His series of inexpensive, modern Hebrew writings—called *sifre agorah* [‘penny literature’] or by the nickname *Ben-Avigdorlech* [‘little Ben-Avigdors’] and published between 1891 and 1892—was aimed at the Jewish masses (Szwabowicz, 2019, pp. 49–50). Ben Avigdor’s idea to reach a wide Jewish audience with popular literature in Hebrew was unprecedented and represented a totally new approach in Hebrew publishing. According to Shulamit Shelhav, “His success heralded a radical rethinking about the titles and distribution of Hebrew texts” (Shelhav, 2010).

Ben-Avigdor’s biggest and most famous individual project was the Tushiyah^14^ publishing house, founded in 1896, which introduced Jewish readers to a wide range of Hebrew texts (both original and translated) through its various series, such as “Biblyotekah Ivrit” [‘Hebrew library’] and “Ha-Biblyotekah Ha-Gedolah” [‘The big library’] (Szwabowicz, 2019, pp. 53–54). Of course, Ben-Avigdor was not the first to offer secular literature for adults or children, but, to a considerable degree, he managed to democratize previously elite-oriented Hebrew culture (Szwabowicz, 2017, p. 241). “Thanks to Ben-Avigdor,” Zeev Gries observes, “Hebrew became a language of daily life for the first time since the Roman empire, a secular language that gave Jews access to the modern world like everyone else” (Gries, 2007, p. 185).

Ben-Avigdor could not have been credited with this achievement if not for yet another strategy aimed at creating a Hebrew readership, namely his focus on publications for young readers. Producing Hebrew literature or periodicals for children virtually meant raising a generation of future customers of Tushiyah and supporters of the Hebrew national revival, which Ben-Avigdor advocated. So, apart from the Tushiyah’s publications for adults, between 1896 and 1899 he also published two series for young readers: one for children and the other for teenagers—which amounted to around 300 booklets altogether (Jakubowicz 1997, p. 77). Ben-Avigdor was well aware of the practical challenges of the Hebrew revival. He understood that in order to *have* Hebrew readers, one must first *educate* them—in some cases, create them practically from scratch. To attain this goal, he published various materials for learning Hebrew (textbooks, dictionaries, anthologies) that could be used for Hebrew education and/or self-education. It would have only been natural for users of these publications, after they had developed sufficient Hebrew reading skills, to buy others of Tushiyah’s books and booklets. In this way they supported Ben-Avigdor’s enterprise not only ideologically, by contributing to the Hebrew revival, but also economically by generating income for his publishing house (Jakubowicz, 1997, pp. 77–78).

As already mentioned, in 1901 Ben-Avigdor started to publish *Olam Katan*, co-edited with Shemu’el Leib Gordon. The original intention of the editors was to make the children’s press more diversified by producing a separate periodical for children and another one for teenagers. However, for practical reasons, in the beginning they had to narrow this grand vision and restrict themselves to a children’s periodical (*OK*, 1901, no. 1, col. [59–60]^15^). It was only in 1903 that they introduced a new press title, *Ha-Ne’urim* [‘Youth’], for teenagers reading in Hebrew. Additionally, in 1902 Tushiyah started to publish *Ha-Pedagog*, a special magazine addressed to Jewish parents and teachers, which was created to offer advice and information on raising Jewish children, Hebrew education, and teachers’ issues (*OK*, 1902, no. 1, col. 23–24). The print runs of all these periodicals remain unknown.

After the closure of *Olam Katan* and *Ha-Ne’urim* in 1904, for reasons which will be discussed later, Ben-Avigdor did not give up on young readers. Apart from the two aforementioned series for young readers published in the final years of the nineteenth century, in the new century the Tushiyah launched three series of literature for young readers, which continued after the closure of the periodicals. The series were aimed at different age groups—from the youngest children to teenagers (*Nitsanim* [‘Buds’], *Peraḥim* [‘Flowers’], and *Bikurim* [‘first fruits’])—and altogether comprised 550 booklets (Ofek, 1979, p. 234). These three series clearly demonstrate not only Ben-Avigdor’s awareness that children’s literature should differ from writings for adults, but also his sensitivity to the specific needs of children at different ages.

This considerable degree of attentiveness to children’s needs distinguished *Olam Katan* from earlier publications and also set a high standard for future Jewish children’s press—as Avraham Levinson observed: *Olam Katan* “in its value and elegance, and also in the extent of its dissemination, rose above all the children’s weeklies which appeared afterwards in the Diaspora” (Levinson, 1935, p. 201). The periodical was also acclaimed for the quality of its linguistic style. One contemporary found its language “as poetic and beautiful as the language of Isaiah, and as simple as a mind of the child” (Melechowicz, 1901, p. 8).^16^

## ***Olam Katan***: F**rom the Idea to the Logistics**

In the first issue of *Olam Katan*, Ben-Avigdor stated that the periodical should bring its readers joy and pleasure in their free time, but also instil moral virtues, develop their aesthetic sensibility and love for nature, and teach them about the world in general, and about the history of their people and the Land of Israel in particular. He hoped that *Olam Katan* would provide proper reading material for the young and thus fill a gap in the education of Jewish children (*OK*, 1901, no. 1, col. [59–60]). The editors believed that the Hebrew language would be revived in the children’s mouths, which means that they expected Jewish children not only to read but also to speak Hebrew (*OK*, 1901, no. 1, col. 6). The role of *Olam Katan* was also to bring up “a generation of Hebrews [i.e. Jews] true in their hearts and souls” (*OK*, 1902, no. 53 col. [43–44]).

In the second year of publishing of *Olam Katan*, there came some substantial changes. First of all, the periodical moved from Vienna to Cracow—to the printing house of the renowned Jewish printer, Josef Fischer. Secondly, the price was reduced, but so was the number of pages. In the first year, subscribers in Russia paid 6 rubles for a one-year subscription, receiving, on average, a 24-page issue each week (or 44 pages in a double issue); in the second year, however, the same subscription cost 4 rubles but the number of pages dropped to 12 or 20, respectively, for a single or double issue, and was followed only by a minimal enlargement of the page size. So, although the subscription prices were reduced, the periodical in fact became more expensive per page. Thirdly, there was a change in the visual style: *Olam Katan* got a new masthead, introduced new decorative elements, and the print quality significantly improved. These changes in form were accompanied by a change in linguistic style; while in the first year only some texts were printed with the *nikud*, in the second year the *nikud* became standard, apart from sections with correspondence and advertisements. All this was supposed to make the periodical more accessible to young readers. Ben-Avigdor hoped that this improved version of *Olam Katan* would awaken the joy of childhood (*gilat ha-yaldut*), which, as he observed, “Jewish children very often lacked” (*OK*, 1902, no. 53, col. [41–44]).

The periodical continued in this form, style, price and frequency for another two full years, during which the editors had to cope with various obstacles connected to its publication. The natural challenge was the political geography: *Olam Katan* was printed first in Vienna, then in Cracow, but the Tushiyah publishing house had its seat in Warsaw, and the majority of the subscribers, as attested to by the readers’ correspondence in *Olam Katan*, lived in the Russian Empire. The logistical complications of mailing a periodical published in the territory of Austria-Hungary to Russia or Poland caused many delays, which must have been equally problematic for the editors, sellers and, of course, readers. The problems with logistics demanded additional expenses and, as a result, generated financial losses. In the fourth and last year of *Olam Katan’*s publication, this situation forced Ben-Avigdor to raise the subscription price (to 7 rubles for a one-year subscription in Russia) and curtail the frequency of the publication; the periodical retained the same number of pages^17^ but appeared only twice per month (*OK*, 1904, no 51–52, col. 1131–1132). However, this solution did not save *Olam Katan*: it was discontinued after the sixth issue, in December 1904. The official reason the editors gave to justify their decision was the appearance of a new magazine for young readers edited by Israel Biniyamin Levner and printed in Vilna under the title *Ha-ḥayim ve-ha-teva* [‘The life and the nature’] (*OK*, 1904, no. 6, col. [201–202]). By the mutual agreement of the editors (Levner was in fact one of Tushiyah’s co-operators), the new periodical took over *Olam Katan*’s subscribers (*OK*, 1904, no. 6, col. [231–232]). Although intended mainly for Russian Jewish children and actually published in Russia, which logistically gave *Ha-ḥayim ve-ha-teva* a better chance of succeeding, *Ha-ḥayim ve-ha-teva* did not last long (see Ofek, 1979, pp. 363–366).

## A Recipe for a Hebrew-Language Children’s Periodical

“The revival of the Hebrew language was not a simple matter, even for its heroes,” Benjamin Harshav observes (1993, p. 86). While undertaking the enterprise of creating a modern illustrated Hebrew weekly for Jewish children, and thus contributing to the Hebrew revival, Ben-Avigdor and Gordon faced several challenges. They had to make the language and content of the periodical accessible for young readers. *Olam Katan* had to combine Hebrew education with national Jewish propaganda, and still appear interesting enough to attract a juvenile audience. To reach a wide audience, the editors had to include both Jewish boys and girls at a time when the latter still had fewer chances for Hebrew education. The Hebrew of the periodical needed to be understood by children who were learning Hebrew as a second or third language^18^. This meant a radical departure from the flowery, Bible-inspired style characteristic of earlier nineteenth-century writings of the Jewish Enlightenment, as well as from the more traditional but equally difficult rabbinical Hebrew. Another challenge was to help Hebrew catch up, after being in a half-dormant state for centuries, with the modern world, so the language could describe or discuss contemporary phenomena. How did Ben-Avigdor and Gordon meet all these challenges? What was their recipe for the Hebrew-language press for children?

Texts in *Olam Katan* presented a wide variety of genres and themes. The ideologically important group of writings spoke of Erets Yisra’el, Zionist endeavours, development of the Jewish settlement in Palestine, Jewish history and tradition, and biographies of famous Jews. Such literature was meant to mould Jewish children into future supporters of the Jewish national cause and exercise their national spirit. Other literary texts, although not free from the didactic agenda, presented narratives about family life, children, and the adventures or misadventures of human or animal characters. Literature promoting popular science revealed secrets of the world of plants, animals and natural phenomena before children’s eyes.

In the literature on popular science, especially, we can observe how *Olam Katan*’s editors and writers coped with the challenge of using the insufficient and unfixed vocabulary of Hebrew. These linguistic struggles are well illustrated by vocabulary relating to various botanical or zoological species. For example, in a text discussing the speed attained by certain animals, a giraffe is called by a descriptive name constructed from existing Hebrew words—*namer-gamal* (‘a leopard camel’) which is an analogy to *cameleopard* in English and in other European languages. Some other animals are referred to using words recycled from the Hebrew Bible: *zarzir motnayim*, a biblical hapax legomenon, designates a sighthound, while a whale is represented by the word *livyatan* (in the Bible—‘Leviathan’, ‘a sea monster’) and, for more precision, it is translated into Yiddish (*walfish*) and to Russian (*kit’*). Names of other animals entered Hebrew as loanwords from other languages and are either explained descriptively in the text or translated into languages which the editors of *Olam Katan* thought their readers would know (mostly Yiddish and Russian). So, an albatross appears with the added information *min tsipor ha-yam* (‘a kind of a sea bird’), and a dolphin is accompanied by *min tanin ha-yam* (‘a kind of a sea-creature [literally: a sea-crocodile]’) (*OK*, 1903 no. 29, col. 635–636). In another text, the aforementioned *tanin ha-yam* designates not a dolphin but a shark, which is additionally described as *min dag toref* (‘a kind of fish of prey’) and translated into Yiddish (*hayfish*) and Russian (*akul’a*) (*OK*, 1902, no. 7, col. 149). The linguistic condition of *Olam Katan* was typical of the state of Hebrew on the eve of revival, which Harshav describes as follows:

(…) Hebrew lacked the simplest words in many domains of life, not just of the modern world but of the basic domestic and surrounding objects: Jews either paid no attention to concrete nature or else used words from other languages. In a multilingual culture, that was not a problem; it could be left either to the spoken language or to the language of the majority population. Hebrew dictionaries, even in the second half of the nineteenth century, translated the most basic names of plants and birds from other languages into Hebrew with: “a kind of tree” or “a kind of bird’. Whenever such terms were needed, foreign names were simply embedded in the Hebrew text (…). (Harshav, 1999, p. 83)

*Olam Katan*, with its texts, documents a fascinating stage in the development of Modern Hebrew, during which the language was a laboratory where new words were (re)invented and experimented with.

How did the editors acquire the literature that filled the columns of *Olam Katan*? It took some time before professional writers of Hebrew-language children’s literature emerged (Shavit, 1996, p. 785). So, in order to provide the periodical with content suitable for young readers, the editors often turned to adaptations and translations. Texts printed in *Olam Katan* thus fall into three categories: (1) texts for children originally written in Hebrew (sometimes especially for the Tushiyah Publishing House), (2) texts adapted from other works written in Hebrew for adults and (3) texts translated/adapted from works in other languages. Authors and translators contributing to *Olam Katan* lived not only in Eastern Europe but also in Palestine (Erets Yisra’el). Some of them were in fact the initiators of the new Hebrew culture in Erets Yisra’el.^19^

The first category (1) may be best represented by the works of Yehudah Steinberg, described as “the Hebrew Andersen,” a prolific author of Hebrew and Yiddish literature who wrote both for youth and adults (see Ofek, 1988, pp. 26–30). His collection of fables, *Ba-ir u-va-ya’ar* (‘In the city and in the forest’), was the first book originally written for children that Tushiyah handled (Bar-El, 2011). Texts of this type were crafted specifically to meet the needs of young readers. Contributors of original Hebrew literature to *Olam Katan* were often relatively young (see Ofek, 1979, p. 361), in their thirties (like Hemdah Ben-Yehudah, Pesach Kaplan, Micha Yosef Berdyczewski, and Yehudah Steinberg), twenties (like Sholem Asch, Ya’acov Fichman, Ya’acov Rabinowitz, and Sha’ul Tschernichowsky) or even younger—Shneour Zalman and Yitshak Katzenelson^20^ were still in their teens when their texts were published in the periodical. Some of these authors, like Sha’ul Tschernichowsky, were already established writers, while others became important figures in the pantheon of Hebrew/Jewish literature only later. As Zohar Shavit indicates, children’s literature was often penned by prestigious writers for adult audiences, who perceived this kind of creativity as their “national task, an indispensable component of the creation of the new nation” (Shavit, 1996, p. 785).

As original children’s literature in Hebrew was still being developed in the studied period, the texts from the second group (2) came in handy for filling in the gap in the readings offered to Jewish children. *Olam Katan* recycled Hebrew texts written for adults, which were rephrased in simple Hebrew and their content adjusted to suit the young readers of the periodical. The sources for these adaptions were manifold: from the Bible to the modern Hebrew press. The news sections especially were often based on other Hebrew periodicals, for example *Ha-Tsefirah*, *Ha-Tsofeh* or *Ha-Melits* (*OK*, 1903, no. 4 col. 81–85).

Another way to increase the volume of children’s literature in Hebrew was to turn to already existing texts in other languages (3). This literature—mostly European and American—was either translated, translated and adapted or loosely paraphrased.^21^ Sometimes translations were made without acknowledging the original author.

Apart from literary texts and articles on religion, history, nature, science, biographical texts, etc., another important part of *Olam Katan* was its news section, entitled *Ḥadashot*, and random information section—*Yedi’ot Shonot*. The first provided young readers with information on recent developments in the world, with a special focus on Eastern Europe, where the majority of the readers lived, and on Palestine. *Ḥadashot* became yet another channel to promote the periodical’s Zionist agenda, but it also had an additional important feature: this section proved that Hebrew could be used to describe what was happening in the modern world and gave readers the feeling that they—like the adults with their regular press—had access to knowledge about the world. Judging from its content, the material intended for the second information section, *Yedi’ot Shonot*, was selected more for its sensational or intriguing character than for its topicality or trustworthiness. Children could read in *Yedi’ot Shonot*, for example, about a journey through the Sahara Desert, giant trees in California, the threat of the Dead Sea’s disappearance, “rain of hay” in England, various uses of coffee in the world, a tombstone erected for a monkey in France, a dog that could read, and a tourist tragedy on Mt. Blanc (*OK*, 1902, nos. 5–6, col. [121–122]). It may puzzle readers of the periodical today that the editors did not shield children from extreme or tragic events. *Olam Katan* published information, for example, about the pogrom in Kishinev in 1903, the war between Russia and Japan that broke out in 1904, natural disasters and personal tragedies. Studying the periodical thus gives us insight into what was considered suitable for children at the beginning of the twentieth century.

One of the important features of *Olam Katan*, which distinguished it from earlier periodicals for children, were illustrations. These served several purposes. They undoubtedly made the periodical visually appealing, which added to its potential to attract readers. The images illustrating fictional texts served as an anchor, helping a reader to better relate to what was written and to focus their attention on the story. Those images accompanying texts on popular science, religion or history also had a didactic purpose; illustrations representing animals, plants, landscapes, famous people, biblical or historical scenes offered a chance to better understand the world beyond the place and time in which the child lived. Some visual content sent a clear ideological message: by documenting the progress of Zionism, it was also meant to agitate for it. For example, to commemorate the Sixth Zionist Congress, issue no. 50 in 1903 included a poster of the Congress’ gathering that was the size of two regular pages (col. [1115–1118]). There were also images intended solely for children’s fun and entertainment, like cartoons and illustrated jokes. Some others had purely decorative purpose.

Some of the images published in *Olam Katan*, like some of the texts, were created specifically for the periodical, but others were taken from external sources. In the first group, for example, were works by Gavri’el Tshorny, who had a long-standing collaboration with the periodical and even designed its second masthead. The problem with the second group was that, unlike text, images could not be easily modified to meet the needs of young Jewish readers. Some of the “recycled” illustrations in *Olam Katan* appear truly peculiar. Let us analyse two examples. In 1901, there appeared an image of a teacher meeting his students, all of them boys, out in the street (*OK*, 1901, no. 13, col. 605–606). The boys in this illustration are evidently not Jewish because, as a sign of respect, they uncover their heads in front of their teacher, which Jewish boys, who are obliged by religious law to cover their heads, would never do. The second example is even more striking. In 1902, there is an image depicting a Jewish father, mother and son with facial features characteristic of… an anti-Semitic caricature (*OK*, 1902, nos. 5–6, col. 123–124). It remains a mystery how this image made it into pages of a periodical for Jewish children.

Another element characterizing *Olam Katan*, and missing from the two chronologically earlier Hebrew periodicals for children, was the regular correspondence section where the editors printed letters from young readers. These letters helped children to relate to each other’s joys and problems, provided role models for young supporters of the Zionist cause, and promoted the idea of learning Hebrew and using it in everyday life. Here the revived language gave the young readers the feeling of being connected to, and of forming, a transregional community of Hebrew users no matter where they lived—in Russia, Palestine, Germany, Romania, Bulgaria or America. Today these letters are invaluable and rare sources, preserving hundreds of children’s voices and helping us to reconstruct their places of residence, ages, genders, family and economic situations, educational patterns, Hebrew levels, reading preferences, etc.^22^ They also point to the role of adults as facilitators of Zionist Hebrew culture for children.

## **The Dual Readership: Children and Adults in*Olam Katan***

As Zohar Shavit wrote, “The most characteristic feature of children’s literature is its double attribution. By definition, children’s literature addresses children, but always and without exception, children’s literature has an additional addressee—the adult, who functions as either a passive or an active addressee of texts written for children” (1999, p. 83). Literature scholars also speak about the concept of the “hidden adult” in children’s literature (Nodelman, 2008) or “dual/double audience” of children’s literature (Bullen & Nichols, 2011; Cheetham, 2013). All these concepts refer, however, to literature for children written in their actively spoken native tongue, which they learn as their first language.

The “double audience” in the case of languages which are re-vernacularized or post-vernacular is more complicated. As Agnieszka August-Zarębska shows in her analysis of contemporary Judeo-Spanish poetry for young readers in the post-vernacular context, “the adult recipient is necessary not only as an intermediary between the child and the text, but also between the child and the language itself” (August-Zarębska, 2021, p. 4). The situation she studies bears both similarities and differences to the case of Hebrew periodicals for children. In *Olam Katan*, published at the outset of the revival of Hebrew as a spoken language and as a first language, the role of the adult as the intermediary between the language and the child is unquestionable. However, in the context of a post-vernacular language it is the generation of the grandparents/parents who speak/understand the language better than the generation of children; in the case of re-vernacularized Hebrew the trend is just the opposite: it is the children’s generation who will use Hebrew to a greater extent and in many more contexts than previous generations. The second difference is gender: in the case of Judeo-Spanish, gender was unrelated to proficiency in the language, while in the case of Hebrew at the beginning of the twentieth century it was. In fact, due to unequal opportunities for traditional Hebrew education, Hebrew was considered to be more of a “father tongue” than a “mother tongue” (Bar-Adon, 2011, p. 541, f. 12), and so the positions of men, women and children in the first stages of the re-vernacularisation of Hebrew were different (Bar-Adon, 1991). Numerous examples of children’s correspondence published in *Olam Katan* confirm that the role of male adults (teachers or family members) as facilitators of Hebrew culture for children^23^ was much more prevalent than in the case of female adults. This situation was still the consequence of the traditional education received by the previous generation, which offered girls fewer opportunities to learn Hebrew than boys.^24^

There is one more aspect of the adult-child relation in the context of the re-vernacularization of Hebrew. In 1903, Hemdah Ben-Yehudah, the second wife of the aforementioned Eli’ezer Ben-Yehudah, published an autobiographical story in *Olam Katan* titled *Le-toldotai* (‘The story of my life’), which she dedicated to her husband who taught her Hebrew. In the story she describes her childhood: how she was born; how people came to see her as an infant and talked to her, but she was unable to speak back to them; how after 13 months she started to grasp what was being said; how after another 13 months she began to speak, and, later, how she learned to read and write. Only towards the end of the text does the reader discover that the story Hemdah narrates did not actually begin the day she was biologically born, but metaphorically – by her “birth” she means leaving Russia and emigrating to Palestine, where she married Eli’ezer Ben-Yehudah and learned Hebrew. Hemdah concludes her story, “I was twenty years old when I was born, and now I am ten” (*OK*, 1903, no. 12, col. 243–246; quotation: col. 244). In this moving and honest confession, Hemdah shows that after emigration, she, an adult Jewish woman, found herself in the Zionist circle in Palestine as speechless as a new-born child, and she compares her process of language acquisition to that of a child. Although she is certainly proud of what she managed to achieve, Hemdah is also painfully aware of her limitations: she is an adult woman, but her Hebrew competence is still at the level of a 10-year-old child.

In this context, a Jewish child who had been raised as a Hebrew speaker, would have a clear advantage over an adult who had to “be born again” in Hebrew. Basmat Even-Zohar notices that Jewish children in Palestine (Erets Yisra’el), although acting within the framework developed by the adults, actively supported the new Hebrew culture, and in the later period became themselves agents of this new culture and facilitated it for the older generation(s) in their family (see Even-Zohar Basmat, 2010, pp. 39, 43). They were expected to lead “the Hebrew revolution” (see Shavit, 2010). This image of the children’s mastery of the language was a vital part of the Zionist narrative, although, as Zohar Shavit has demonstrated, the reality of the Yishuv (Jewish settlement in pre-state Israel) was much more multilingual than this narrative seemed to suggest (see Shavit, 2017, 2021).

## Conclusion

*Olam Katan* was not the first periodical intended for Jewish children reading in Hebrew, but it was probably the first one that achieved some measure of success, judging from the time span of its publication, the authors who published there, and the enthusiastic letters from its readers. Children’s literature in general, and *Olam Katan* in particular, was one of the most strategic means by which Ben-Avigdor aspired to create Hebrew mass readership. Jewish children and teenagers were offered writing in modern Hebrew, which they read at home for pleasure and used as study materials in different types of Jewish schools. The periodical established clear-cut role models for children, who were expected to grow up as Hebrew users and supporters of the Jewish national movement.

Yael Reshef reminds readers of her study that “The revival of Hebrew as a spoken language was devised from the outset to be carried out by the younger generation” (2021, p. 20). And although she and other scholars (e.g. Bar-Adon, 2011; Shavit, 2010) emphasize the role of children in the process of the revival of Hebrew in Palestine, it is also important to remember the role of children in the Diaspora. Jewish children in Eastern Europe were critical to the emergence of the young Hebrew-reading public. Moreover, as is attested to by their correspondence, children in the Diaspora engaged in using Hebrew beyond a passive reading competence: they wrote letters, stories and poems, and attempted to speak Hebrew not only with adults but also between themselves, both at school and beyond it.

Ben-Avigdor’s dream of contributing to the Hebrew revival by creating young Hebrew readers may have started with *Olam Katan*, but it certainly did not end with it. His publishing empire provided children and youth who wished to read or speak Hebrew with accessible literature, periodicals and educational materials. *Olam Katan*, however, was much more than just one example of printed Hebrew material published by the Tushiyah. By means of this periodical, Ben-Avigdor and Gordon managed to create a network of readers throughout Russia and Poland, but also reaching Palestine, and far beyond. They influenced the group identity of young readers and provided them with a platform that brought this transregional community closer.

## Footnotes

1 On the rise and development of literature for Jewish children in the eighteenth and/or nineteenth centuries, see for example Kogman (2016, 2019) and Shavit (1988, 1994).

2 This periodical should not be confused with *Olam Katon* published in Jerusalem in 1893, nor with *Olami*, *Olami Ha-katan*, *Olami Ha-ketantan* published in the 1930s in Warsaw. The transcription of the word *shevu’i* in the subtitle (and not *shavu’i*, as in the original title) is dictated by the decision to follow the standard Modern Hebrew transcription in this article.

3 For more biographical information about each of them, see respectively: Shelhav (2010) and Holtzman (2010).

4 The literature on the Hebrew revival is extensive. For a brief and concise introduction to the subject (with references for further reading), see Reshef (2011); on the role of children in the process of the re-vernacularisation of Hebrew, see Even-Zohar Basmat (2020) and Reshef (2021).

5 There were some earlier attempts to reach Jewish children with periodical publications in Hebrew, but they were either quite ephemeral or not even proper magazines. See Ofek (1979, pp. 344–345).

6 For more on gender differentiation in Jewish education in Eastern Europe in the nineteenth century, see Stampfer (1992); on what it meant to “know Hebrew” in Eastern Europe in the nineteenth century, see Stampfer (1993).

7 Although Ben-Yehudah became the symbol of the Hebrew revival, and his emigration from Russia to Palestine in 1881 marks the symbolic beginning of Modern Hebrew, the current scholarship offers new readings of the re-vernacularisation of Hebrew. See, for example, Reshef (2011) and Shavit (2017, 2021).

8 The Second Aliyah was the second wave of Jewish migration to Palestine in 1904–1914, mainly from Russia and Poland.

9 In Modern Hebrew—‘playground’.

10 Ofek notes that a part of *Gan Sha’ashuim* was actually addressed more to teenagers than children, judging from the subjects it discussed. For a more detailed presentation of *Gan Sha’ashuim* see Ofek (1979, pp. 351–359).

11 The information about 85 issues is given following Ofek (1979, p. 353). I was able to consult only 78 issues. Issues of both periodicals, *Olam Katon* and *Gan Sha’ashuim*, are available online at the website of the Historical Jewish Press: https://www.nli.org.il/en/discover/newspapers/jpress (accessed July 14, 2021).

12 The majority of issues of *Olam Katan* from years 1902–1904 are available online via the Jagiellonian Digital Library: https://jbc.bj.uj.edu.pl/dlibra/publication/399962?language=en#structure (accessed April 14, 2022). The issues from the first year of publication can be found in paper copies in the National Library in Warsaw and in the National Library of Israel in Jerusalem.

13 Drawing on Frenk’s opinion about Ben-Avigdor, Magda Sara Szwabowicz interestingly explores the concept of the publisher as the Creator—see Szwabowicz (2017, pp. 244–249).

14 The Hebrew word *tushiyah* can be translated as ‘wisdom’ or ‘understanding’.

15 *Olam Katan* consequently will be abbreviated in the references to: *OK*. The periodical used column numbers instead of page numbers. Unnumbered columns will be reconstructed in the square brackets, as in the example above.

16 I thank Alicja Maślak-Maciejewska for drawing my attention to this source.

17 The regular number of pages for a double issues in 1904 was 20. The only exception in the fourth year of the publication was the last issue (no. 6) which had only 16 pages.

18 The multilingual reality characterized not only the situation of Jewish children in Eastern Europe. In Palestine at this time even if there were children who spoke Hebrew as the main language, often it was not their only language, as we are reminded by Zohar Shavit—“even children and young people who spoke Hebrew nonetheless often conducted parts of their life not in Hebrew and were bilingual or even multilingual in various situations” (Shavit, 2017, p. 116).

19 To this group belonged, for example, Ḥemdah Ben-Yehudah, Yitsḥak Epstein, Yehudah Grazowski-Gur, and Yosef Meyuḥas whose original and/or translated texts appeared in *Olam Katan*. For the activities of this group, see Even-Zohar Basmat (2010, pp. 40–43). For more on the emergence of a native Hebrew culture in Palestine in pre-state Israel, see Even-Zohar Itamar (1990).

20 Uriel Ofek writes that the poem *Ha-sha’on make*, which Yitshak Katzenelson published in *Olam Katan* when he was 16 years old, was actually written at the time of his *bar mitsva* (around age 13). See Ofek (1979, p. 361). The poem was published in *Olam Katan*, 1902, no. 1, col. 1–2.

21 On the status of translated literature in pre-state Israel, see Shavit (1998), and on the translation of children’s literature especially, see Shavit (1981).

22 Although children’s letters printed in *Olam Katan* are a fascinating source, their comprehensive analysis is beyond the scope of this article—I will analyze them in another study.

23 See for example OK, 1901 no. 27, col. 37.

24 The situation of Jewish girls in Palestine (Erets Yisra’el) was also ambivalent. Analyzing the Zionist project of Girls’ School in Jaffa, Yehudit Shteiman showed that girls and their education were treated somewhat instrumentally: “it was worthwhile investing in their education not in order to develop their personalities, but rather in order for them to educate their children better” (Shteiman, 2002, p. II). Paradoxically, this way of seeing girls in the roles of “future mothers” added importance to their education not only in the family sphere but also at the national level (idem.)

## References

[CR1] Assaf David, Etkes Immanuel, Assaf David (2010). ‘Katan ve-ḥamim’? - Ha-shir ‘Oyfn pripetshik’ ve-ha-shinui be-dimuyav shel ha-ḥeder. Ha-ḥeder: meḥkarim, te’udot, pirke sifrut u-zikhronot.

[CR20] August-Zarębska, Agnieszka. (2021). Contemporary Judeo–Spanish poetry for young readers. *Children Literature in Education*. Retrieved April 14, 2022, from https://link.springer.com/article/10.1007/s10583-021-09464-7

[CR36] Bar-Adon Aaron, Kaye Alan S. (1991). Semitic studies in honor of Wolf Leslau, on the occasion of his eighty-fifth birthday, November 14th, 1991.

[CR21] Bar-Adon, Aaron. (2011). On the role of children in the revival of Hebrew. In William C. McCormack and Stephen A. Wurm (Eds.), *Approaches to language: Anthropological issue* (pp. 531–552). Berlin, New York: De Gruyter Mouton. Retrieved April 4, 2022, from https://www.degruyter.com/document/doi/10.1515/9783110800036.531/pdf

[CR22] Bar-El, Adina. (2011). Children’s literature: Hebrew literature (Transl. C. Friedman-Cohen). In *YIVO encyclopedia of Jews in Eastern Europe*. Retrieved March 8, 2022, from https://yivoencyclopedia.org/article.aspx/Childrens_Literature/Hebrew_Literature

[CR2] Bullen Elizabeth, Nichols Susan (2011). Dual Audiences, Double Pedagogies: Representing Family Literacy as Parental Work in Picture Books. Children’s Literature in Education.

[CR3] Cheetham Dominic (2013). Audience in children’s literature. English Literature and Language.

[CR23] Even-Zohar, Basmat. (2010). Shituf ha-yeladim ba-yozmah ha-‘ivrit ba-shanim 1880–1905. In *Yeladim be-rosh ha-maḥaneh: yaldut u-neurim be-‘itot mashber u-temurah* (= *Dor le-dor* 36), 39–69.

[CR4] Even-Zohar Itamar (1990). The Emergence of a Native Hebrew Culture in Palestine, 1882–1948. Poetics Today.

[CR24] Frenk, A[zriel] N[atan (Nosn)]. (1916). Ben-Avigdor: La-sifrut ha-‘ivrit ma pa’al?, In *Ben-Avigdor: Le-ḥag yovlo bi-melot ‘esrim ve-ḥamesh shanah la-‘avodato ha*-*sifrutit ve-ha-molit**(1891–1916)*. Varshah.

[CR25] Gries, Zeev. (2007). *The book in the Jewish world, 1700–1900*. Oxford-Portland, OR: The Littman Library of Jewish Civilization. Retrieved May 20, 2021, from https://search.ebscohost.com/login.aspx?direct=true&db=nlebk&AN=1863666&lang=pl&site=eds-live

[CR5] Harshav Benjamin (1993). Language in Time of Revolution.

[CR26] Holtzman, Avner. (2010). Gordon, Shemu’el Leib (Trans: David Fachler). In *YIVO encyclopedia of Jews in Eastern Europe*. Retrieved April 14, 2022, from https://yivoencyclopedia.org/article.aspx/Gordon_Shemuel_Leib

[CR27] Jakubowicz, Bernard. (1997). ‘Asot va-ḥafec sefarim harbeh. Le-toldoteha shel hotsa’at ha-sefarim „Tsentral”-„Merkaz” be-Varshah (1911–1933). Unpublished MA Thesis, Tel Aviv University.

[CR6] Kogman Tal (2016). The emergence of scientific literature in Hebrew for children and youth in the nineteenth century: Preliminary directions for research. Jewish Culture and History.

[CR7] Kogman Tal (2019). Texts for boys and for girls: Concepts of childhood, gender and education in German Jewish Society in the eighteenth and nineteenth centuries. Journal of Jewish Studies.

[CR28] Levinson, Avraham. (1935). *Ha-tenu’ah ha-‘ivrit ba-golah*. Varshah.

[CR29] Melechowicz J (1901). Olam Katan. Wschód.

[CR8] Nodelman Perry (2008). The hidden adult: Defining children’s literature.

[CR9] Ofek Uriel (1979). Sifrut ha-yeladim ha-‘ivrit ha-ḥadashah – ha-hatḥalah.

[CR10] Ofek Uriel (1988). Sifrut ha-yeladim ha-‘ivrit, 1900–1948.

[CR30] *Olam Katan.* (1901–1904). Shemu’el Leib Gordon, Avraham Leib Shalkovich (Eds.). Vienna-Cracow: Tushiyah.

[CR11] Reshef Yael, Weninger Stefan (2011). The re-emergence of Hebrew as a national language. The semitic languages: An international handbook.

[CR12] Reshef Yael, Helman Anat (2021). The role of children in the revival of Hebrew. No small matter: Features of Jewish childhood.

[CR13] Shavit Zohar (1981). Translation of children’s literature as a function of its position in the literary polysystem. Poetics Today.

[CR14] Shavit Zohar (1988). From Friedländer’s Lesebuch to the Jewish Camp—The beginning of Hebrew children’s literature in Germany. Leo Baeck Yearbook.

[CR31] Shavit Zohar (1994). Deutsche Einflüsse auf jüdische Kinderliteratur. Ariel.

[CR15] Shavit Zohar, Hunt Peter (1996). Hebrew and Israeli children’s literature. International companion encyclopedia of children’s literature.

[CR32] Shavit, Zohar. (1998). The status of translated literature in the creation of Hebrew literature in pre-state Israel (the *Yishuv* Period). *Meta, 43*(1), 1–8. Retrieved July 25, 2022, from https://id.erudit.org/iderudit/004128ar

[CR16] Shavit Zohar, Beckett Sandra L. (1999). The double attribution of texts for children and how it affects writing for children. Transcending boundaries writing for a dual audience of children and adults.

[CR17] Shavit Zohar (2010). Yeladim ke-nos’e mahpekhat ha-dibur ha-‘ivrit. Dor Le-Dor.

[CR18] Shavit Zohar (2017). "Can It Be That Our Dormant Language Has Been Wholly Revived?“: Vision, propaganda, and linguistic reality in the Yishuv under the British mandate. Israel Studies.

[CR37] Shavit Zohar (2021). Ma dibru ha-yeladim ha-‘ivriyim? Al proyekt ha-‘ivri’ut u-veni’at ḥevrah le’umit be-Erets Yisra’el. Israel.

[CR33] Shelhav, Shulamit. (2010). Shalkovich, Avraham Leib (Trans: David Fachler). In *YIVO encyclopedia of Jews in Eastern Europe*. Retrieved April 14, 2022, from https://yivoencyclopedia.org/article.aspx/Shalkovich_Avraham_Leib

[CR34] Shteiman, Yehudit. (2002). ‘Itsuv demuto shel ha-yeled ha-‘ivri he-ḥadash ke-ḥelek mi-tikhnun tarbut be-Erets Yisra’el be-reshit ha-me’ah ha-20. Doctoral Thesis, Tel Aviv University.

[CR35] Stampfer Shaul (1992). Gender differentiation and education of the Jewish woman in nineteenth-century Eastern Europe. Polin.

[CR38] Stampfer Shaul, Glinert Lewis (1993). What did “knowing Hebrew” mean in Eastern Europe?. Hebrew in Ashkenaz: A language in exile.

[CR39] Szwabowicz Magda Sara (2017). Wydawcy w służbie idei. O pionierach hebrajskiego świeckiego ruchu wydawniczego. Studia Judaica.

[CR19] Szwabowicz Magda Sara (2019). Hebrajskie życie literackie w międzywojennej Polsce.

